# High Levels of Circulating Monocytic Myeloid-Derived Suppressive-Like Cells Are Associated With the Primary Resistance to Immune Checkpoint Inhibitors in Advanced Non-Small Cell Lung Cancer: An Exploratory Analysis

**DOI:** 10.3389/fimmu.2022.866561

**Published:** 2022-04-13

**Authors:** Giuseppe Bronte, Elisabetta Petracci, Serena De Matteis, Matteo Canale, Ilaria Zampiva, Ilaria Priano, Paola Cravero, Kalliopi Andrikou, Marco Angelo Burgio, Paola Ulivi, Angelo Delmonte, Lucio Crinò

**Affiliations:** ^1^ Department of Clinical and Molecular Sciences (DISCLIMO), Università Politecnica delle Marche, Ancona, Italy; ^2^ Unit of Biostatistics and Clinical Trials, Istituto di Ricovero e Cura a Carattere Scientifico (IRCCS) Istituto Romagnolo per lo Studio dei Tumori (IRST) “Dino Amadori”, Meldola, Italy; ^3^ Unit of Immunobiology of Transplants and Advanced Cellular Therapy, Istituto di Ricovero e Cura a Carattere Scientifico (IRCCS) Azienda Ospedaliero-Universitaria di Bologna, Bologna, Italy; ^4^ Biosciences Laboratory, Istituto di Ricovero e Cura a Carattere Scientifico (IRCCS) Istituto Romagnolo per lo Studio dei Tumori (IRST) “Dino Amadori”, Meldola, Italy; ^5^ Section of Oncology, Department of Medicine, University of Verona; Centro Ricerche Cliniche di Verona (CRC), Verona, Italy; ^6^ Department of Medical Oncology, Istituto di Ricovero e Cura a Carattere Scientifico (IRCCS) Istituto Romagnolo per lo Studio dei Tumori (IRST) “Dino Amadori”, Meldola, Italy

**Keywords:** myeloid-derived suppressive cells, primary resistance, immunotherapy, non-small cell lung cancer, immune checkpoint inhibitors

## Abstract

**Background:**

Immunotherapy has become the standard of care for non-small cell lung cancer (NSCLC) patients. Some patients experience primary resistance to immunotherapy. Currently, we lack a marker of resistance to immunotherapy. Myeloid-derived suppressive-like cells (MDSCs) can reduce tumor response rate and survival outcomes.

**Methods:**

This is an exploratory prospective observational study on metastatic NSCLC patients starting immunotherapy. Baseline peripheral blood samples were collected. Monocytic (M)-MDSCs were analyzed by flow cytometry. The main clinical outcomes were tumor response, progression-free survival (PFS), and overall survival (OS). The association between MDSC levels and tumor response was assessed. The association of PFS with OS was investigated using the Kaplan–Meier method and the Cox proportional hazards model.

**Results:**

Twenty-two patients were included. The median M-MDSC value was higher in patients with progressive disease than patients with stable disease or partial response, p = 0.045. The median MDSC value in the overall population was 1.9. We found worse PFS (HR = 2.51; p = 0.046) and OS (HR = 2.68; p = 0.042) in patients with M-MDSC values higher than the median.

**Conclusions:**

In this exploratory analysis, high M-MDSC levels are strongly associated with primary resistance to immunotherapy. If validated in larger studies, MDSC levels in blood samples could help to select NSCLC patients for higher benefit from immunotherapy.

## Introduction

Lung cancer remains the leading cause of cancer-related death, even though new therapeutic options are available nowadays. The advent of immune checkpoint inhibitors (ICIs) contributed to radically change the therapeutic scenario for non-small cell lung cancer (NSCLC). The currently approved ICIs usually inhibit programmed death 1 (PD-1) and its ligand (PD-L1). PD-L1 is a cell surface protein, and it can be expressed in numerous tissues. It causes anergy or apoptosis binding PD-1, which is expressed on the surface of cytotoxic T cells (1). Tumor cells express PD-L1 as a mechanism of immune escape (2). The suppression of the PD1/PD-L1 axis induces an antitumor immune response *via* the inhibition of the mechanisms of immune tolerance.

In the last few years, before the approval of chemoimmunotherapy as the upfront treatment in metastatic NSCLC patients, pembrolizumab was used as the first-line treatment for a PD-1 expression >50% and in the subsequent lines of treatment for PD-L1 > 1% (3, 4). Conversely, the other ICIs nivolumab and atezolizumab were approved for subsequent lines of treatment regardless of PD-L1 expression (5–9).

To date, we need a deeper knowledge about the underlying mechanisms of immune escape and therapeutic resistance to ICIs that occur in the majority of patients, leading to tumor progression (10). One important resistance mechanism is installed by the acquisition by NK and T cells of an exhausted phenotype, and the immunosuppressive function of regulatory T cells (Tregs) and myeloid-derived suppressive-like cells (MDSCs) (11).

MDSCs are considered an obstacle for many cancer immunotherapies. MDSCs are immature myeloid cells, which can increase as a result of chronic inflammation because of soluble and exosome-bound factors produced by the tumor. MDSC includes 2 major subsets: polymorphonuclear (PMN-MDSC) and monocytic (M-MDSC) (12). A high number of MDSCs have been observed in patients with NSCLC, breast cancer, and head and neck cancer (13). Increased levels of circulating MDSCs have recently been correlated with disease stage and extensive metastatic tumor burden in patients with breast cancer (14). MDSCs suppress the immune system, mainly T-cell function, through the reduction of antigen-specific CD8^+^ T-cell proliferation and the increase of T-cell apoptosis. Some authors hypothesized that the elimination of MDSCs may significantly improve antitumor response and enhance the efficacy of cancer immunotherapy (15–17). Specifically, Terabe et al. highlighted that the depletion of CD11b^+^/Gr-1^+^ cells abrogated TGF-β production and consequently prevented tumor recurrence in mice (15). Similarly, Kusmartsev et al. reduced the presence of immature myeloid cells through all-trans-retinoic acid in mice, and consequently, they improved the tumor-specific CD4- and CD8-mediated immune response (17). Overall, the strategies to target MDSCs include the following: depleting MDSC populations, blockade of MDSC migration, attenuating MDSC immunosuppressive functions, and inducing MDSC differentiation. These strategies could also be combined with other immunotherapies (18, 19).

The present exploratory study aims to investigate if immune cell populations measured in peripheral blood samples before ICI treatment are associated with the primary resistance to these drugs.

## Material and Methods

### Study Population

This is a prospective observational exploratory study on patients diagnosed with advanced NSCLC who received an ICI according to clinical practice at IRST IRCCS between November 2018 and December 2019.

To be included, these patients had to meet the following inclusion criteria: 1) patients with histological or cytological diagnosis of locally advanced (primary or recurrent) or metastatic NSCLC candidate to an immunotherapy treatment with a PD-1 or a PD-L1 inhibitor alone or in combination with other ICIs; 2) participants are willing and able to give informed consent for participation in the study; and 3) measurable disease with at least 1 measurable lesion.

In agreement with Response Evaluation Criteria in Solid Tumors (RECIST) v1.1, tumor response was classified as complete response (CR), partial response (PR), stable disease (SD), and progressive disease (PD). CR is the disappearance of all target lesions and any pathological lymph nodes with a reduction in short axis of less than 10 mm. PR means ≥30% decrease in the sum of diameters of target lesions, taking as reference the baseline sum diameters. PD means ≥20% increase in the sum of diameters of target lesions, taking as reference the smallest sum observed, and an absolute increase ≥5 mm, or the appearance of one or more new lesions. SD includes neither sufficient reduction to be classified as PR nor sufficient increase as for PD.

The study was reviewed and approved by the Ethics Committee of IRST and Area Vasta Romagna (CE.ROM., Study Code B093; Prot. 7658/2018 I.5/233, October 17, 2018). Informed consent was obtained from each patient for use of biological material for research purposes. The study complied with the provisions of the Good Clinical Practice guidelines and the Declaration of Helsinki.

### Sample Collection and Flow Cytometric Analysis

Samples of peripheral blood (15–20 ml in EDTA tubes) were collected before ICI was started (baseline). The analysis of M-MDSC was performed on fresh whole blood until 4 h from sample collection. The following mAbs were used after red blood cell lysis for M-MDSC identification: CD11b, CD14, CD15, CD33, CD45, and HLA-DR. The antibodies were all from Miltenyi Biotec (Bergisch Gladbach, Germany). The percentage of M-MDSC on CD45^+^ cells was obtained. As reported in **Supplementary Figure 1**, the CD14^+^HLA-DR^−/low^ cell subset was gated, and the proportion of CD11b^+^CD33^+^ was evaluated (20).

In addition, a lymphocyte gate was set based on the CD45 and SSC parameters. Gating strategy was used to identify exhausted CD3^+^, CD56^+^, and CD3^+^CD56^+^ cell subsets by evaluating the expression of PD-1 or LAG-3 as previously reported (17). Tregs were defined by the expression of CD25 and FoxP3 and low expression of CD127 among CD4^+^ T cells.

Flow cytometric analysis was performed using a 2-laser FACS Canto (BD Biosciences, San Jose, CA, USA). A minimum of 100,000 CD45^+^ lymphocytes were recorded for each analysis. Flow cytometry data were analyzed with DiVa 6.1.1 software. Appropriate isotype controls were included for each sample. The antibodies were all from Miltenyi Biotec.

### Statistical Analysis

Data were summarized by median, interquartile (IQ) range, and minimum and maximum values for continuous variables and through natural frequencies and percentages for categorical ones.

The association between categorical variables was tested by Pearson’s χ^2^ test or Fisher’s exact test, when appropriate, whereas those between a continuous variable and a categorical one were tested by means of the Mann–Whitney U test or the Kruskal–Wallis test, as appropriate. Correlation among variables was measured by means of Spearman’s coefficient.

The studied time-to-event endpoints were progression-free survival (PFS) and overall survival (OS). PFS was defined as the time from the start of immunotherapy to disease progression or death for any cause, whichever occurred first. Patients who were alive and progression-free on September 30, 2021, the last follow-up update, were censored at that date. OS was defined as the time from the start of immunotherapy to death from any cause. Alive patients were censored at the date of the last follow-up update. PFS and OS functions were estimated using the Kaplan–Meier method, and the log-rank test was used to assess differences between groups. Median PFS and OS were reported as point estimates and 95% CIs in round brackets. The median follow-up time was determined using the reverse Kaplan–Meier method. The Cox proportional hazards regression model was used to quantify the association between specific covariates and the time-to-event endpoints. Results are reported as HR and 95% CI in round brackets.

Overall and when not otherwise specified, a two-sided p-value (p) <0.05, was considered statistically significant. All statistical analyses were performed using STATA 15.0 software (College Station, TX, USA) and R version 4.1.0.

## Results

Twenty-two patients met eligibility criteria and signed informed consent. The baseline patient characteristics are summarized in **Table 1**. Male patients were 68.2%, and the median age was 70 years (range: 55–82). All patients but one were diagnosed with lung adenocarcinoma (95.5%). The majority of the patients were former or current smokers (81.8%). Almost half of the patients received atezolizumab (45.5%), while the others received pembrolizumab, nivolumab, or a combination of ICIs. More than a half underwent second-line immunotherapy, but just a few patients received immunotherapy as third-line therapy. Two-thirds of patients who received second- or third-line immunotherapy had platinum-based chemotherapy as a first-line treatment. All patients were tested for druggable oncogene drivers, i.e., EGFR, ALK, and ROS1. All of them were found to be non-oncogene addicted.

**Table 1:** Patients’ baseline characteristics (n = 22).

	n	(%)
**Gender**		
F	7	(31.8)
M	15	(68.2)
**Age at start of immunotherapy**		
Median [IQ range]	70.1 [64.8–75.0]	
**Histotype**		
Adenocarcinoma	21	(95.5)
Other	1	(4.6)
**Smoking habit**		
Never	4	(18.2)
Former	13	(59.1)
Current	5	(22.7)
**Type of immunotherapy**		
Pembrolizumab	5	(22.7)
Nivolumab	3	(13.6)
Atezolizumab	10	(45.5)
Combination	4	(18.2)
**Line at start of immunotherapy**		
First	6	(27.3)
Second	15	(68.2)
Third	1	(4.6)
Type of first-line chemotherapy agent*		
Platinum-based	14	(87.5)
Non-platinum-based	2	(12.5)

n, number; F, female; M, male; IQ, interquartile.

^*^For patients receiving immunotherapy since the second line.

We obtained data on absolute blood cell count at baseline including neutrophils, lymphocytes, monocytes, and platelets. From these parameters, we calculated the systemic inflammation indicators (SII): neutrophil-to-lymphocyte ratio (NLR), platelet-to-lymphocyte ratio (PLR), and lymphocyte-to-monocyte ratio (LMR). The phenotypic characterization of other immune cell subpopulations was evaluated by flow cytometry at baseline. The median value and range for each cell subset are reported in **Supplementary Table 1**.

No patients experienced a CR. Four patients (18.2%) achieved a PR, 6 patients (27.3%) had an SD, and 12 patients (54.5%) experienced a PD as the best response. The median duration of response was 2.0 months (IQ range: 1.9–3.7).

**Figure 1:**
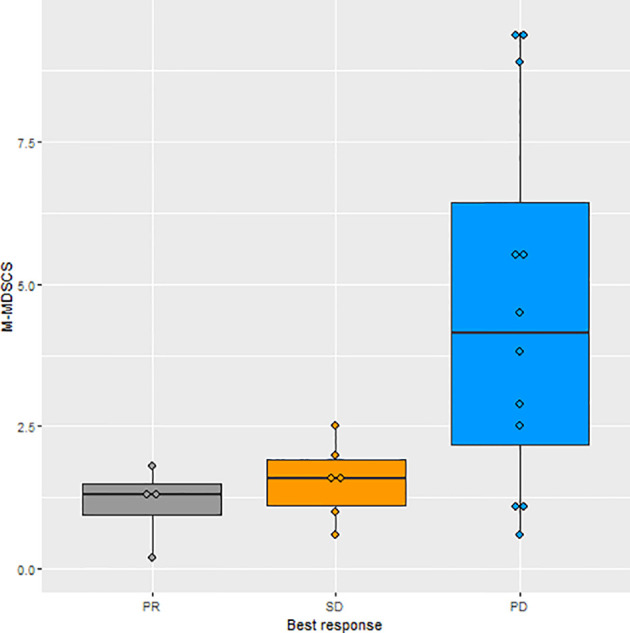
Boxplot of the distributions of M-MDSC levels in relation to best tumor response. M-MDSC, monocytic myeloid-derived suppressive-like cell.

**Figure 2:**
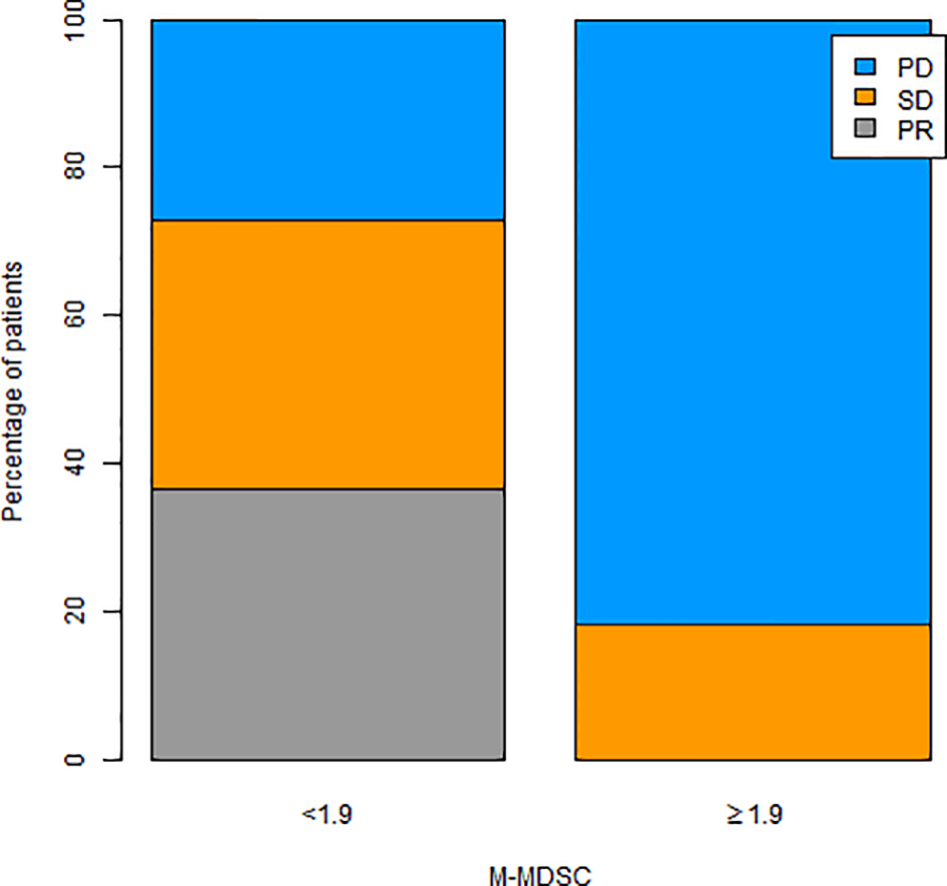
Tumor response according to M-MDSC levels (< vs. > median value). M-MDSC, monocytic myeloid-derived suppressive-like cell.

When the levels of all the immune cell subtypes were analyzed with respect to tumor response, only M-MDSCs showed a statistically significant association. Specifically, the patients who experienced PD at the first imaging evaluation had higher levels of M-MDSCs at baseline (median: 4.2; IQ range: 1.9–7.3) than patients with PR (median: 1.3; IQ range: 0.7–1.6) or SD (median: 1.6; range: 1.0–2.0), p = 0.045 (**Figure 1**). Of patients with higher M-MDSC levels, 81.8% reported a PD as the best response, whereas 18.2% with higher M-MDSC levels reported an SD. The difference between the groups was statistically significant (p = 0.022) (**Figure 2**). The only 4 PR observed among the 22 patients had M-MDSC levels lower than the median. The associations between other-than-MDSC cell subpopulations and radiological response are depicted in **Supplementary Figure 2**.

**Figure 3:**
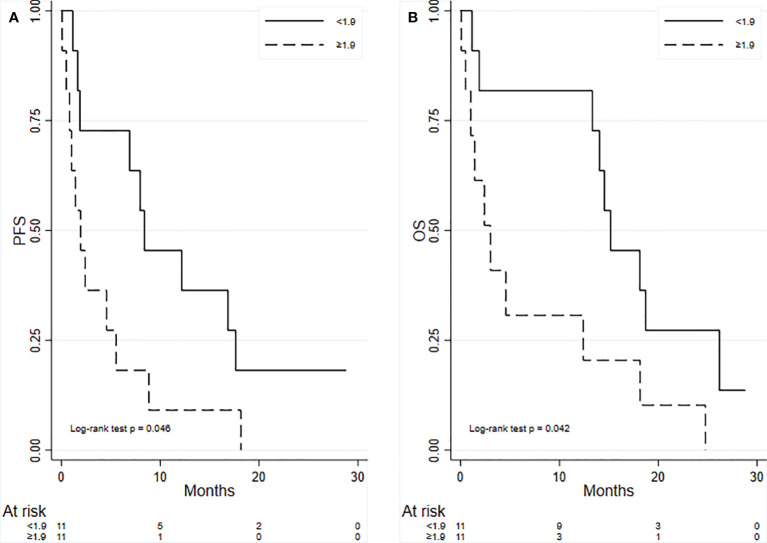
Kaplan–Meier curves for the analysis of PFS **(A)** and OS **(B)** according to M-MDSC levels (< vs. > median value). PFS, progression-free survival; OS, overall survival; M-MDSC, monocytic myeloid-derived suppressive-like cell.

In the overall population, the median PFS was 4.6 months (95% CI: 1.41–8.85), whereas the median OS was 13.3 months (95% CI: 1.88–18.13). **Table 2** shows the results from univariate analyses for PFS according to all baseline covariates (**Table 2**). With regard to NLR and PLR, higher values were associated with worse PFS; the opposite was true for LMR. M-MDSC levels equal or greater than 1.9 were associated with shorter median PFS as compared to lower values: 1.94 months (95% CI: 0.49–5.53) vs. 8.39 months (95% CI: 1.64–17.63); HR: 2.51 (95% CI: 1.02–6.19), p = 0.046 (**Figure 3A**).

**Table 2:** Univariate analysis for PFS.

	HR (95% CI)	p
**Gender**		
F	1 (ref)	
M	1.01 (0.39–2.63)	0.987
**Age at start of immunotherapy**	1.02 (0.95–1.08)	0.630
**Histology**		
Adenocarcinoma	1 (ref)	
Other	2.46 (0.30–20.05)	0.400
**Smoking habit**		
Never	1 (ref)	
Former	0.85 (0.27–2.71)	0.782
Current	0.69 (0.17–2.82)	0.608
**Type of immunotherapy**		
Pembrolizumab	1 (ref)	
Nivolumab	12.35 (1.90–80.21)	0.008
Atezolizumab	2.37 (0.64–8.81)	0.198
Combination	1.74 (0.37–8.26)	0.483
**Line at start of immunotherapy**		
First	1 (ref)	
Second	0.79 (0.29–2.14)	0.637
Third	1.00 (0.12–8.52)	0.998
**M-MDSC**		
<1.9	1 (ref)	
≥1.9	2.51 (1.02–6.19)	0.046
**Neutrophils** ^*^	1.81 (1.14–2.89)	0.012
**Lymphocytes** ^*^	0.58 (0.33–1.02)	0.059
**Platelets** ^*^	1.49 (0.95–2.33)	0.080
**Monocytes** ^*^	2.74 (1.49–5.02)	0.001
**NLR** ^*^	2.94 (1.57–5.50)	0.001
**PLR** ^*^	2.66 (1.43–4.95)	0.002
**LMR** ^*^	0.26 (0.12–0.57)	0.001
**CD3^+^** ^*^	1.00 (0.68–1.46)	0.995
**CD56^+^** ^*^	0.93 (0.63–1.38)	0.730
**CD4^+^PD-1^+^** ^*^	1.08 (0.72–1.62)	0.694
**CD3^+^PD-1^+^** ^*^	1.16 (0.81–1.64)	0.418
**CD56^+^PD-1^+^** ^*^	1.04 (0.70–1.57)	0.830
**CD3^+^LAG-3^+^** ^*^	1.15 (0.67–1.96)	0.621
**CD56^+^LAG-3^+^** ^*^	0.91 (0.61–1.36)	0.643
**Tregs** ^*^	0.92 (0.60–1.41)	0.697
**M-MDSC** ^*^	1.74 (1.10–2.74)	0.018

HR, hazard ratio; p, p-value; F, female; M, male; ref, reference; NLR, neutrophil-to-lymphocyte ratio; PLR, platelet-to-lymphocyte ratio; LMR, lymphocyte-to-monocyte ratio; Tregs, regulatory T cells; M-MDSC, monocytic myeloid-derived suppressive cells; SD, standard deviation.

^*^Variables are reported as 1 − SD unit increase.

Similar results were obtained with respect to OS (**Table 3**). M-MDSC levels higher than the median value 1.9 were associated with worse median OS: 3.03 months (95% CI: 0.49–12.40) vs. 15.16 months (95% CI: 1.88–26.18); HR: 2.68 (95% CI: 1.04–6.95), p = 0.042 (**Figure 3B**).

**Table 3:** Univariate analysis for OS.

	HR (95% CI)	p
**Gender**		
F	1 (ref)	
M	1.13 (0.40–3.23)	0.814
**Age at start of immunotherapy**	1.05 (0.98–1.11)	0.160
**Histology**		
Adenocarcinoma	1 (ref)	
Other	3.53 (0.41–30.24)	0.251
**Smoking habit**		
Never	1 (ref)	
Former	0.89 (0.28–2.89)	0.851
Current	0.72 (0.17–2.98)	0.653
**Type of immunotherapy**		
Pembrolizumab	1 (ref)	
Nivolumab	8.52 (1.38–52.53)	0.021
Atezolizumab	3.69 (0.85–15.96)	0.198
Combination	1.05 (0.23–4.73)	0.951
**Line at start of immunotherapy**		
First	1 (ref)	
Second	1.22 (0.45–3.35)	0.698
Third	1.26 (0.14–11.05)	0.836
**M-MDSC**		
<1.9	1 (ref)	
≥1.9	2.68 (1.04–6.95)	0.042
**Neutrophils** ^*^	1.91 (1.18–3.09)	0.008
**Lymphocytes** ^*^	0.44 (0.23–0.84)	0.012
**Platelets** ^*^	1.31 (0.87–1.98)	0.198
**Monocytes** ^*^	2.19 (1.24–3.86)	0.007
**NLR** ^*^	3.23 (1.68–6.19)	<0.001
**PLR** ^*^	2.89 (1.52–5.50)	0.001
**LMR** ^*^	0.16 (0.06–0.41)	<0.001
**CD3^+^** ^*^	0.92 (0.68–1.38)	0.674
**CD56^+^** ^*^	1.00 (0.66–1.52)	0.996
**CD4^+^PD-1^+^** ^*^	1.12 (0.75–1.66)	0.591
**CD3^+^PD-1^+^** ^*^	1.40 (0.95–2.07)	0.086
**CD56^+^PD-1^+^** ^*^	1.06 (0.72–1.56)	0.776
**CD3^+^LAG-3^+^** ^*^	1.27 (0.72–2.24)	0.401
**CD56^+^LAG-3^+^** ^*^	1.02 (0.67–1.55)	0.937
**Tregs** ^*^	0.85 (0.54–1.32)	0.466
**MO-MDSC** ^*^	1.97 (1.19–3.26)	0.008

HR, hazard ratio; p, p-value; F, female; M, male; ref, reference; NLR, neutrophil-to-lymphocyte ratio; PLR, platelet-to-lymphocyte ratio; LMR, lymphocyte-to-monocyte ratio; Tregs, regulatory T cells; M-MDSC, monocytic myeloid-derived suppressive cells; SD, standard deviation.

^*^Variables are reported as 1 − SD unit increase.

**Figure 4:**
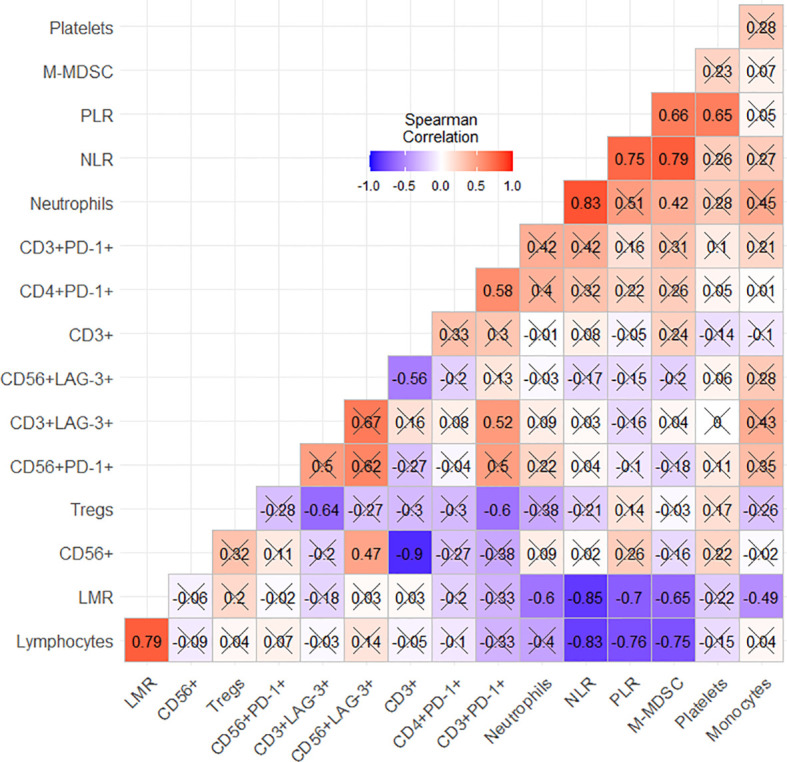
Spearman’s correlation coefficients. “X” refers to a not statistically significant correlation (p < 0.05).

**Figure 5:**
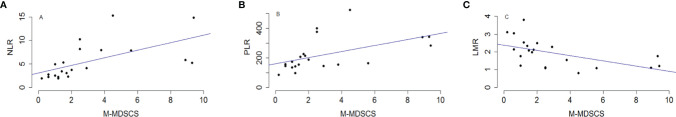
Scatter plots with regression lines as regards the correlation between M-MDSCs and SIIs, NLR **(A)**, PLR **(B)**, and LMR **(C)**. M-MDSC, monocytic myeloid-derived suppressive-like cell; SIIs, systemic inflammation indicators; NLR, neutrophil-to-lymphocyte ratio; PLR, platelet-to-lymphocyte ratio; LMR, lymphocyte-to-monocyte ratio.

Finally, the correlation between immune cell subtypes and the inflammatory indexes was also explored (**Figure 4**). We found a strong statistically significant positive correlation between M-MDSCs and NLR (ρ = 0.79; p < 0.001) and PLR (ρ = 0.66; p = 0.001). These correlations might make us suppose that higher M-MDSCs could be related to systemic inflammation. The scatter plots with the regression line highlight this potential interplay (**Figure 5**).

## Discussion

The search for biomarkers of primary resistance to ICIs led us and other researchers to explore the role of circulating immune cells, given that data on PD-L1 and tumor mutational burden (TMB) are not fully consistent for patients’ selection. For this reason, we mainly analyzed the baseline levels of exhausted T and NK lymphocytes, Tregs, and M-MDSCs. Among these cell subsets, only high levels of circulating M-MDSCs seem to be associated with primary resistance to ICIs, even though the sample size was small.

The effect of MDSCs on the reduction of antitumor response by immune cells has been known for a long time. MDSCs can reach tenfold levels in circulating blood from patients with various malignancies, favoring neoangiogenesis and metastases (21, 22). In NSCLC patients, M-MDSCs were more frequent than normal monocytes in both tumor tissue and lymph nodes, but the highest levels were observed in the peripheral blood. In all NSCLC tissues, M-MDSCs produced a higher amount of TGF-beta (23). However, some authors analyzed circulating PMN-MDSCs at baseline in patients with advanced NSCLC and found higher levels in those experiencing a better response under the treatment with nivolumab as second-line therapy (24). This finding is opposite to ours about M-MDSCs and lets us suppose that these two subsets of MDSCs can exert different roles as regards the resistance to ICIs. In a similar population of NSCLC patients treated with pembrolizumab or nivolumab, Feng et al. found that those who experienced a PR to immunotherapy had higher levels of M-MDSCs at baseline. This finding is also opposite to ours. The authors of this work sought to explain this result with a supposed effect of anti-PD-1 treatment on the decrease of M-MDSCs, but they state that “the mechanism for the decreased M-MDSCs after anti-PD-1 treatment in PR group was unknown” (25). Conversely, in another work, NSCLC patients who showed low levels of PMN-MDSCs or M-MDSCs at baseline experienced a longer PFS and a better OS, and a decrease or increase of these cells in the peripheral blood was not associated with changes in PFS and OS. Interestingly, the authors found that low circulating IL-6 levels correlated with better PFS and that IL-6, but not TGF-beta, was associated with M-MDSCs (26). This finding supports the role of some immunosuppressive cytokines in mediating the negative effects of M-MDSCs on antitumor immune response. A different scenario was reported by Youn et al. (27), where circulating PMN-MDSCs at baseline were not different between responder and non-responder NSCLC patients treated with nivolumab. However, after the first cycle of anti-PD-1 therapy, these cells were lower among responders than among non-responders. Concurrently, NK cells showed an opposite behavior. They found that the NK cell-to-PMN-MDSC ratio was predictive and prognostic, achieving a 0.866 accuracy (27). Similarly, Kim et al. (28) described no significant differences in the frequency of PMN-MDSCs at baseline between responders and non-responders in a study in which they explored the Tregs-to-PMN-MDSC ratio. PMN-MDSCs are largely represented in peripheral lymphoid organs. M-MDSCs are more present in the tumor microenvironment, where rapidly they differentiate to tumor-associated macrophages. For this reason, these subpopulations should be considered separately, and we discussed the studies on PMN-MDSCs to show the controversial results on this topic (29). All these studies on circulating MDSCs showed that these cells can alter the efficacy of immunotherapy in NSCLC, even though the findings may appear conflicting. Perhaps the small number of patients investigated in these studies was limited to achieve an unambiguous result, as the samples of patients ranged between 27 and 63. We hypothesized that studying a wider population of NSCLC patients treated with the same therapy could help to reach clearer findings. Moreover, we are aware that there are some limitations in correlating the data on immune monitoring by flow cytometry and the data on routine blood cell count. However, this correlation could be useful to make this analysis close to the clinical practice.

Some researchers developed strategies to alter MDSC functions and consequently overcome immunotherapy resistance. Some chemotherapeutics have already been known to reduce the frequency of MDSCs in the tumor microenvironment (30, 31). However, in advanced NSCLC patients treated with first-line chemotherapy, bevacizumab-based regimens more significantly reduced the levels of PMN-MDSCs in peripheral blood than those not containing bevacizumab (32). This result can be explained by the effect of VEGF on MDSC differentiation and expansion (33). This hypothesis is supported by the significant effect of a combination of sunitinib, a multikinase VEGF receptor inhibitor, with nivolumab on MDSC reduction in the tumor microenvironment from a renal cell carcinoma mouse model (34). Accordingly, in a phase Ib/II trial with sunitinib plus nivolumab in soft tissue sarcoma patients, higher PFS and OS were observed in comparison with nivolumab monotherapy in other studies (35, 36). Another interesting strategy under investigation to reduce MDSC-mediated immunosuppression is gemtuzumab ozogamicin, an antibody against CD33 conjugated to a cytotoxic agent. The researchers observed a greater proliferation of T cells when co-cultured with MDSCs treated with this conjugated anti-CD33 antibody than those co-cultured with untreated MDSCs (37). Finally, we mention further potential strategies that could limit MDSC-mediated immunosuppression, and these include the poly(ADP-ribose) polymerase inhibitor olaparib, IL-12, CCR2 blockade (38).

All these findings discussed here suggest that MDSCs can have an influence on the tumor response and survival outcomes so that the study of this cell subset in the peripheral blood could go on to identify a biomarker of resistance to ICIs. This could be useful not just to select patients that could really benefit from anti-PD-1/PD-L1 therapy but also to identify those patients for whom primary resistance could be overcome through targeting MDSCs. Anyway, the effect of MDSCs on the resistance to ICIs could be different when this kind of immunotherapy is combined with cytotoxic agents because these are known to reduce MDSCs in the tumor microenvironment. Given that recently platinum-based chemotherapy combined with pembrolizumab has become the standard upfront therapy for metastatic NSCLC patients, circulating MDSC levels should be detected also in these patients to verify whether these cells can also influence these new treatment regimens.

## Data Availability Statement

The raw data supporting the conclusions of this article will be made available by the authors, without undue reservation.

## Ethics Statement

The studies involving human participants were reviewed and approved by the Ethics Committee of IRST and Area Vasta Romagna (CE.ROM.). The patients/participants provided their written informed consent to participate in this study.

## Author Contributions

Conceptualization: GB, SDM, and LC. Methodology: EP and SDM. Validation: GB, PU, AD, and LC. Formal analysis: EP, SDM, and MC. Investigation: all authors. Resources: GB, PC, MB, AD, and KA. Data curation: IP, IZ, and GB. Writing—original draft preparation: EP, SDM, and GB. Writing—review and editing: all authors. Supervision: PU, GB, AD, and LC. Project administration: PU, AD, LC, and GB. All authors have read and agreed to the published version of the manuscript.

## Conflict of Interest

The authors declare that the research was conducted in the absence of any commercial or financial relationships that could be construed as a potential conflict of interest.

## Publisher’s Note

All claims expressed in this article are solely those of the authors and do not necessarily represent those of their affiliated organizations, or those of the publisher, the editors and the reviewers. Any product that may be evaluated in this article, or claim that may be made by its manufacturer, is not guaranteed or endorsed by the publisher.

## Supplementary Material

Supplementary Figure 1 Gating strategy of M-MDSC in a patient who obtained PR and a patient in PD.

Supplementary Figure 2 The associations between other-than-MDSC cell subpopulations and radiological response.
