# Supplementary figures and images for: High Levels of Circulating Monocytic Myeloid-Derived Suppressive-Like Cells Are Associated With the Primary Resistance to Immune Checkpoint Inhibitors in Advanced Non-Small Cell Lung Cancer: An Exploratory Analysis
Source: Front Immunol. 2022 Apr 13;13:866561. doi: 10.3389/fimmu.2022.866561 (PMC9043492; doi:10.3389/fimmu.2022.866561)

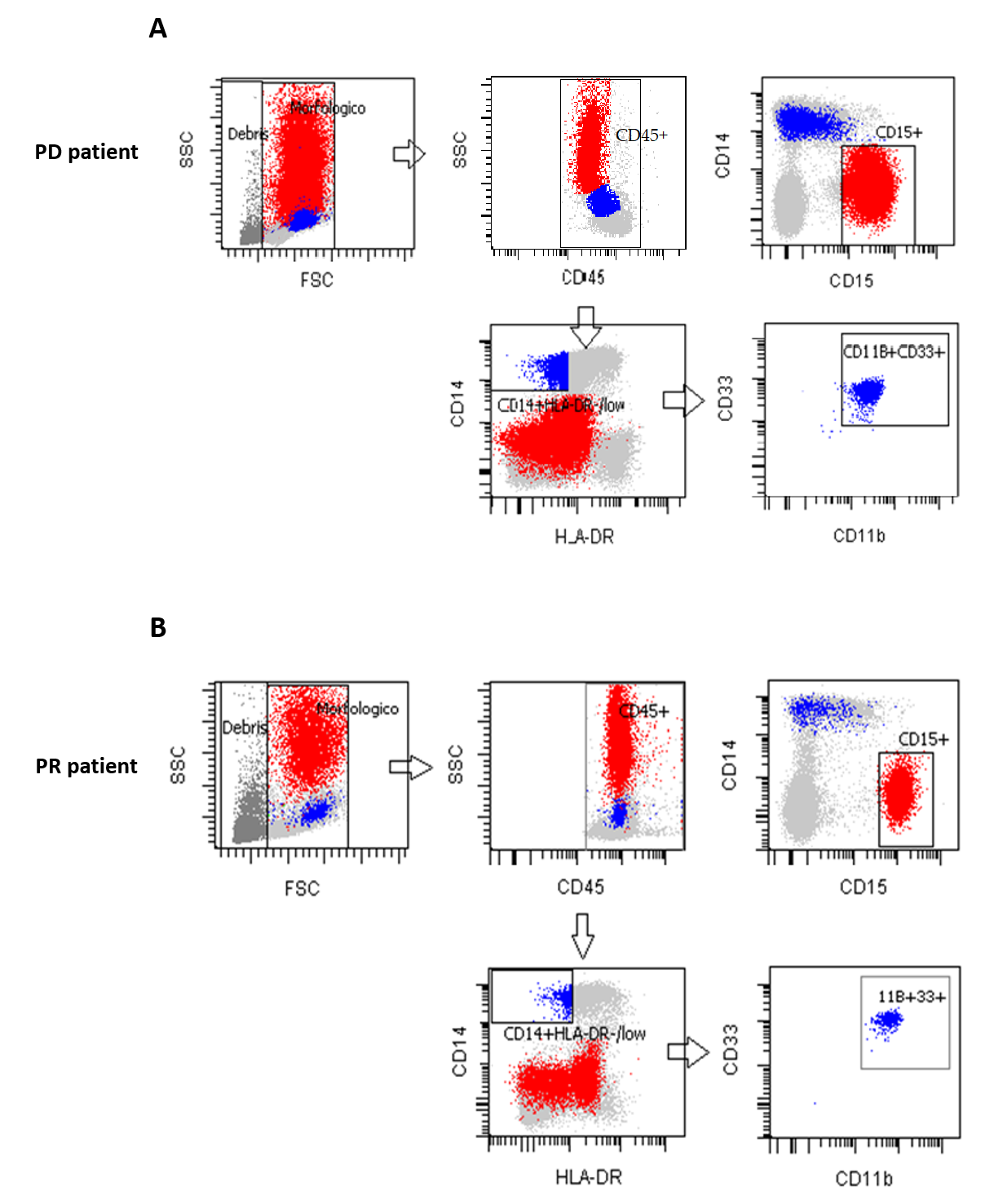

Supplement: Supplementary Figure 1 — Gating strategy of M-MDSC in a patient who obtained PR and a patient in PD. [file Image_1.tif]

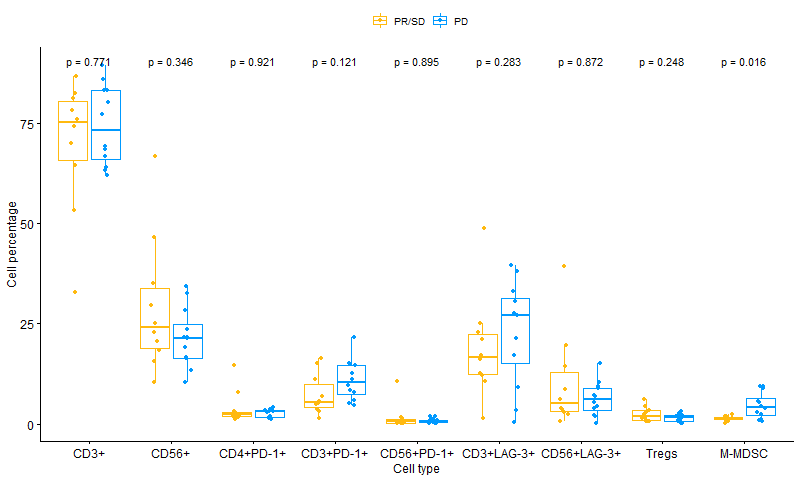

Supplement: Supplementary Figure 2 — The associations between other-than-MDSC cell subpopulations and radiological response. [file Image_2.tif]
