# Supplementary material for: High Levels of Circulating Monocytic Myeloid-Derived Suppressive-Like Cells Are Associated With the Primary Resistance to Immune Checkpoint Inhibitors in Advanced Non-Small Cell Lung Cancer: An Exploratory Analysis
Source: Front Immunol. 2022 Apr 13;13:866561. doi: 10.3389/fimmu.2022.866561 (PMC9043492; doi:10.3389/fimmu.2022.866561)
Supplement: Supplementary file 3 [file Table_1.docx]

| **Neutrophils** |  |  |
| --- | --- | --- |
| Median [IQ range] | 6060 [4500 - 8550] | |
| Min - max | 2960 - 16920 | |
| **Lymphocytes** |  |  |
| Median [IQ range] | 1360 [1140 - 2020] | |
| Min - max | 590 – 2360 | |
| **Platelets** |  | |
| Median [IQ range] | 292500 [227000 - 337000] | |
| Min - max | 158000 - 433000 | |
| **Monocytes** |  | |
| Median [IQ range] | 780 [660 - 940] | |
| Min - max | 480 - 1380 | |
| **NLR** |  | |
| Median [IQ range] | 4.1 [2.5 – 7.8] | |
| Min - max | 1.9 – 15.3 | |
| **PLR** |  | |
| Median [IQ range] | 172.3 [145.2 – 283.3] | |
| Min - max | 83.7 – 523.7 | |
| **LMR** |  | |
| Median [IQ range] | 2.0 [1.2 – 2.3] | |
| Min - max | 0.8 – 3.8 | |
| **CD3+** |  | |
| Median [IQ range] | 75.2 [64.4 – 82.5] | |
| Min - max | 32.8 – 89.5 | |
| **CD56+** |  | |
| Median [IQ range] | 21.7 [16.5 – 29.5] | |
| Min - max | 10.3 – 66.6 | |
| **CD4+PD-1+** |  | |
| Median [IQ range] | 2.5 [1.8 – 3.3] | |
| Min - max | 1.2 – 14.6 | |
| **CD3+PD-1+** |  | |
| Median [IQ range] | 8.2 [5.1 – 14.6] | |
| Min - max | 1.4 – 21.7 | |
| **CD56+PD-1+** |  | |
| Median [IQ range] | 0.7 [0.2 – 1.2] | |
| Min - max | 0.1 – 10.6 | |
| **CD3+LAG-3+** |  | |
| Median [IQ range] | 21.2 [12.2 – 27.6] | |
| Min - max | 0.3 – 48.8 | |
| **CD56+LAG-3+** |  | |
| Median [IQ range] | 5.8 [2.9 – 9.5] | |
| Min - max | 0.1 – 39.4 | |
| **Tregs** |  | |
| Median [IQ range] | 1.7 [0.6 – 2.6] | |
| Min - max | 0.1 – 6.2 | |
| **M-MDSC** |  | |
| Median [IQ range] | 1.9 [1.2 – 4.5] | |
| Min - max | 0.2 – 9.4 | |
| IQ: interquartile; NLR: neutrophil-to-lymphocyte ratio; PLR: platelet-to-lymphocyte ratio; LMR: lymphocyte-to-monocyte ratio; Tregs: T regulatory cells; M-MDSC: monocytic myeloid-derived suppressive cells | | |

**Supplementary Table1.** Descriptive of the inflammatory indexes and immune cell populations
